# Atomic-like spin noise in solid-state demonstrated with manganese in cadmium telluride

**DOI:** 10.1038/ncomms9121

**Published:** 2015-09-18

**Authors:** S. Cronenberger, D. Scalbert, D. Ferrand, H. Boukari, J. Cibert

**Affiliations:** 1 Laboratoire Charles Coulomb UMR 5221 CNRS/UM2, Université Montpellier, Place Eugene Bataillon, Montpellier Cedex 05 34095, France; 2 Université Grenoble Alpes, Grenoble F-38000, France; 3 CNRS, Institut NEEL, Grenoble F-38000, France

## Abstract

Spin noise spectroscopy is an optical technique which can probe spin resonances non-perturbatively. First applied to atomic vapours, it revealed detailed information about nuclear magnetism and the hyperfine interaction. In solids, this approach has been limited to carriers in semiconductor heterostructures. Here we show that atomic-like spin fluctuations of Mn ions diluted in CdTe (bulk and quantum wells) can be detected through the Kerr rotation associated to excitonic transitions. Zeeman transitions within and between hyperfine multiplets are clearly observed in zero and small magnetic fields and reveal the local symmetry because of crystal field and strain. The linewidths of these resonances are close to the dipolar limit. The sensitivity is high enough to open the way towards the detection of a few spins in systems where the decoherence due to nuclear spins can be suppressed by isotopic enrichment, and towards spin resonance microscopy with important applications in biology and materials science.

## Abstract (web summary)

Coupling an atomic vapour to the polarization of light has enabled the creation of spin squeezed and entangled atomic states. Here, the authors realize a solid-state approach by adapting spin noise spectroscopy to probe the spin of manganese ions in cadmium telluride.

## Graphical abstract

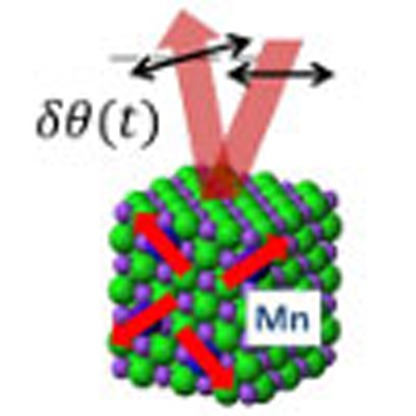

Coupling polarized light to atomic spin ensembles non resonantly, the atom-light interface^1^,^2^, is a powerful approach to manipulate collective spin states, and to generate spin squeezed^3^,^4^ and entangled atomic states^5^. Atomic ensembles, with which these methods have been developed, are hardly scalable. Scalability is more straightforwardly achieved in solids, however such an approach has been limited to carriers in semiconductor heterostructures^6^,^7^,^8^,^9^,^10^,^11^,^12^.

Here, we introduce the atom-exciton-light interface in solids, to transfer the atomic spin noise to light polarization in a system where the atomic spin is not directly coupled to light: in the case of Mn in CdTe which we use as a testbed, the exciton mediates the coupling between atoms and light. This interface is based on the sp-d exchange interaction, which couples the manganese ions to the carriers. Thus atomic spin fluctuations produce tiny splittings of the excitonic transitions, ultimately detected by the induced circular birefringence (Kerr rotation). We show that the spin noise spectra keep their atomic nature despite the strong hybridization between the orbitals of manganese ion and the crystal. This opens new possibilities by transposing in solids the method of quantum physics, based on the atom-light interface.

## Results

### Spin Hamiltonian and the spin noise spectra

Electron spin resonances can be described by a spin Hamiltonian, in which the allowed terms are dictated by symmetry considerations. For Mn in a tetrahedral crystal field it takes the form^13^


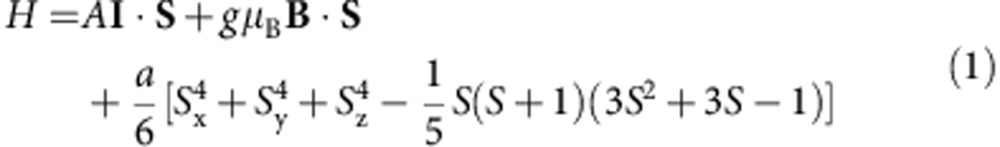


Here **I** and **S** are the Mn nuclear and electronic spins respectively, *μ*_B_ is the Bohr magneton and **B** is the magnetic field. The Hamiltonian is composed of the hyperfine term, with *A*=−170.5 MHz, the Zeeman term, with *g*=2 the Mn *g*-factor, and the cubic crystal field term, with *a*=89.5 MHz (ref. 14).

**Figure 1:**
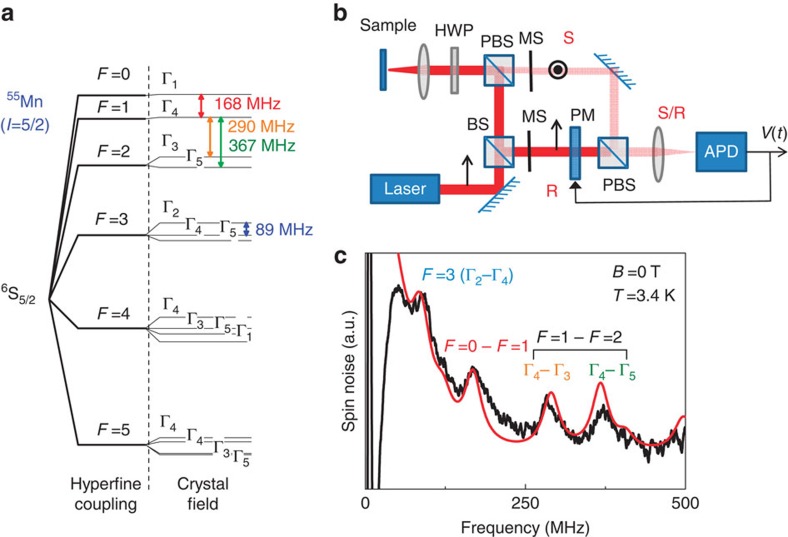
Spin noise of ^55^Mn diluted in bulk CdTe. (**a**) Energy levels of ^55^Mn split by the hyperfine coupling and the cubic crystal field. Arrows point the transitions observed in **c**. (**b**) Experimental setup. A vertically polarized laser beam is focused on the sample. Mn spin fluctuations impart Kerr rotation noise on the reflected probe which is split into two mutually orthogonal polarizations (beam S and R) by a polarizing beamsplitter (PBS). The beam S carries the spin fluctuations while beam R carries only the intensity fluctuations. Both beams are sent alternatively on the avalanche diode (APD) using mechanical shutters (MS), and kept at the same intensity with a feedback loop on the phase modulator (PM). (**c**) Spin noise spectra in zero magnetic field, as measured (black line) and calculated (red line) with only one parameter 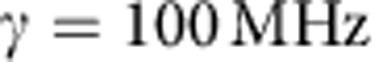. The dip in the experimental spectrum below 50 MHz comes from subtraction of a zero frequency line not accounted for by theory (see text).

The ^6^*S*_5/2_ Mn ground state is split by the hyperfine coupling in six F-levels (Fig. 1a). Without crystal field, the F-levels are split by a magnetic field into 2*F*+1 Zeeman levels, with a common *g*-factor *g*_*F*_=1 because *S*=*I*. The crystal field further splits and mixes the hyperfine levels, which considerably increases the number of allowed transitions^13^.

According to the fluctuation-dissipation theorem the spectrum of the spin fluctuations for a single Mn spin in the direction of the unit vector 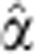, at thermal equilibrium, is related to the susceptibility and is given by^15^


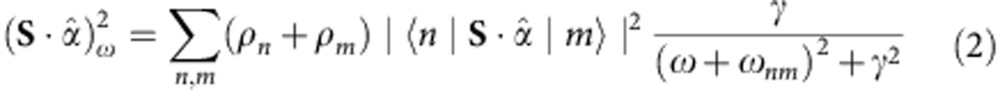


where the double summation is over the (2*S*+1) × (2*I*+1)=36 eigenstates of the spin hamiltonian, *ρ*_*n*_ is the occupation factor at thermal equilibrium of level *n* with eigenfrequency 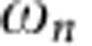 and 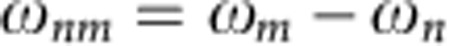. For simplicity, we assume Lorentzian lines with a broadening parameter 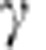, common to all transitions, and written as 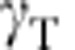 for the transverse configuration 
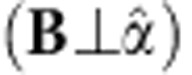
 and 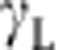 for the longitudinal geometry 
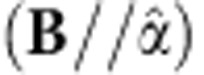
.

In the transverse configuration, Zeeman transitions within each hyperfine level contribute to a single line at *g*_*F*_=1, and inter-hyperfine transitions between adjacent *F*-levels contribute to higher frequency lines. The sum rules derived in Supplementary Note 1 shows that half of the integrated spin noise is concentrated in the *g*_*F*_=1 line, the other half being shared between all inter-hyperfine transitions. In the longitudinal case, 
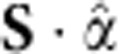
 is diagonal within each hyperfine level and the corresponding noise signal appears around zero frequency. The crystal field brings in some complexity but does not affect significantly the intensity share between intra- and inter-hyperfine transitions.

### Samples and experiment

All the results are obtained from Cd_0.999_Mn_0.001_Te samples: a bulk crystal cleaved along a (110) plane and quantum wells (QW) grown either on the (001) plane of CdTe or Cd_96_Zn_0.04_Te substrates. The spin fluctuations are probed along the laser beam, perpendicular to the sample surface. More details about the samples and the experimental setup (Fig. 1b) can be found in section Methods.

### Bulk sample

**Figure 2:**
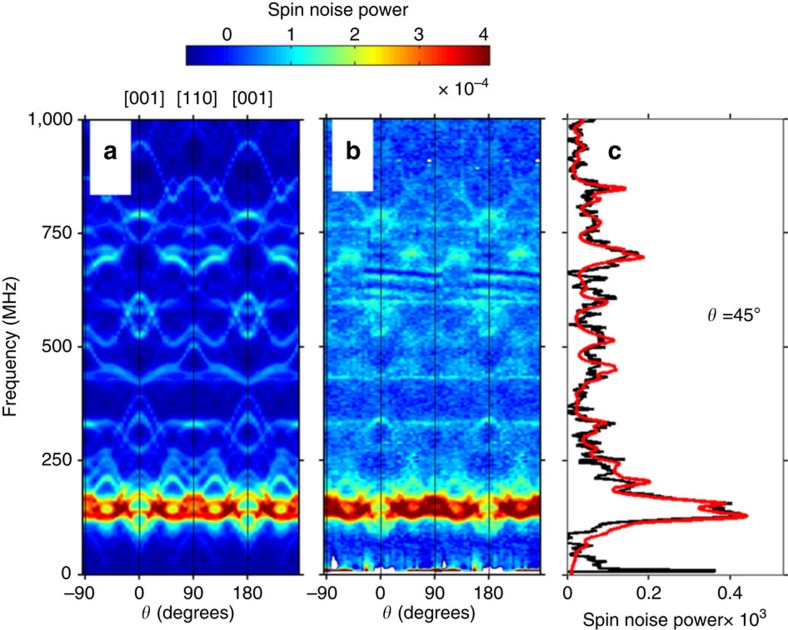
Angular resolved spin noise of ^55^Mn diluted in bulk CdTe at *B*_T_=10.5 mT. (**a**) Contour plot of the spectra calculated with Equation (2) versus *θ*, the angle between magnetic field and the [001] direction contained in the (110) plane. (**b**) Experimental contour plot (*T*=4.8 K). (**c**) Experimental spectrum at *θ*=45° (black line) and best fit (red line) with 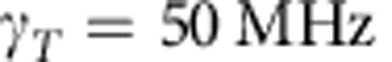.

The spin noise spectrum of Mn diluted in the bulk crystal is reported in Fig. 1c. The four strongest resonances are identified as intra-hyperfine, and inter-hyperfine transitions between states of the Mn split by the cubic crystal field (Fig. 1a). The data are well reproduced by the model with as only fitting parameter the HWHM in omega units 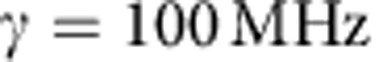. Spin noise spectra have also been measured with a magnetic field applied in all directions of the (110) plane defined by the angle *θ* (by steps of 5°). As expected, we obtain a twofold symmetry in the frequency map. We then average the noise spectra measured at *θ* and *θ*+180° in order to better resolve the weak structures, which spread over the 1 GHz width (Fig. 2b,c). All the dominant features predicted by equation (2) can be recognized in the experimental spectra and many details are perfectly reproduced by the simulation with the linewidth 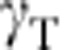 being the only fitting parameter. A line centred at zero frequency, not predicted by the theory, has been subtracted from the experimental spectra to allow the comparison with theory: the original spectra are given in Supplementary Fig. 1.

### Quantum wells

The spin noise spectra are quite sensitive to small lattice distortions. The presence of a lattice mismatch between the QW and substrate imposes adding a spin anisotropy term 
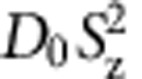
 to the spin hamiltonian. This term is clearly observed in the QW grown on Cd_96_Zn_0.04_Te, with the expected value *D*_0_=+473 neV obtained using the known Mn spin-lattice coefficients^14^. However, the fourfold cubic symmetry is broken. Adding an in-plane anisotropy term partially reproduces the experimental spectra (see Supplementary Fig. 2).

**Figure 3:**
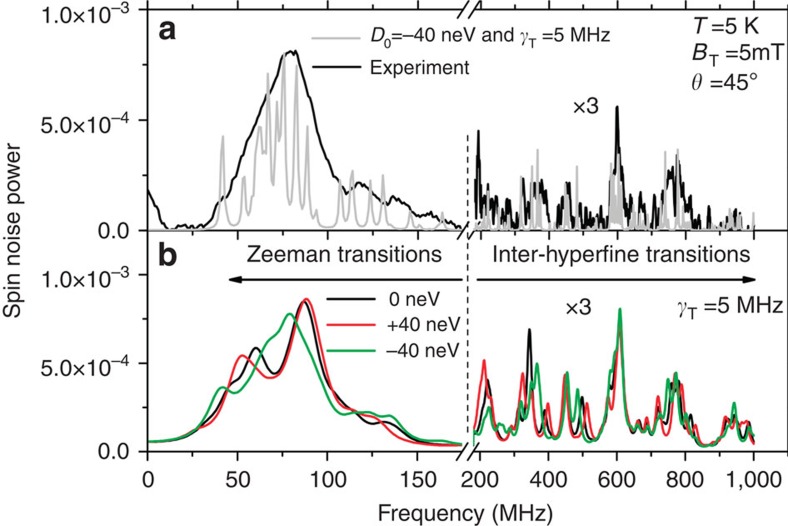
Adjustment of the biaxial strain to fit the experimental spin noise spectra of ^55^Mn in the QW grown on CdTe. (**a**) Experimental (black line) and calculated (grey line) spin noise spectra. As shown by the calculation with a small broadening 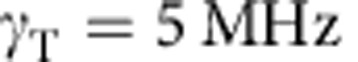, many unresolved individual lines, which belong either to the Zeeman transitions (left) or to inter-hyperfine transitions (right), are unresolved in the experimental spectrum. (**b**) Calculated noise spectra for different values of the strain parameter *D*_0_: the best agreement with the experiment is obtained for *D*_0_=−40 neV and 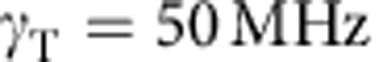 (green curve).

**Figure 4:**
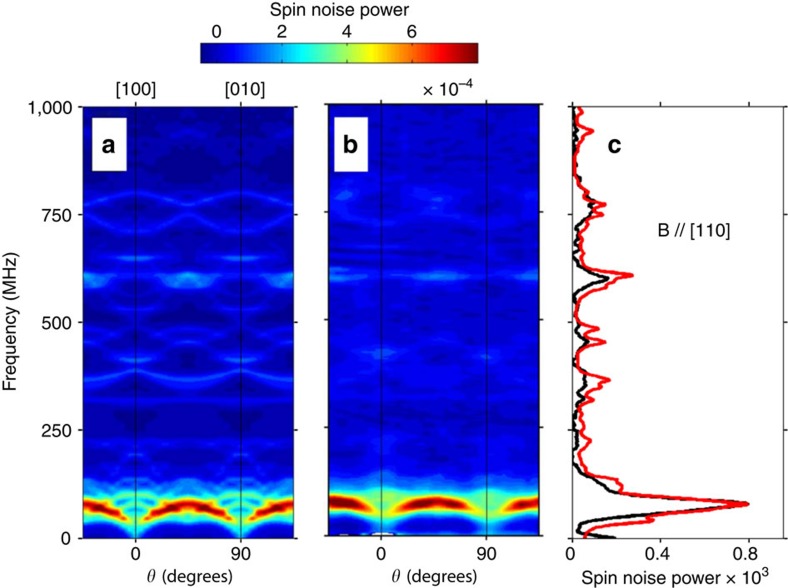
Angular resolved spin noise of ^55^Mn in the QW grown on CdTe at *B*_T_=5 mT. (**a**) Contour plot of spin noise spectra calculated for 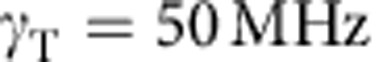, and *D*_0_=−40 neV. (**b**) Experimental contour plot (*T*=5 K). (**c**) Experimental (black line) and calculated spectra (red line) at *θ*=45° (B//[110]).

**Figure 5:**
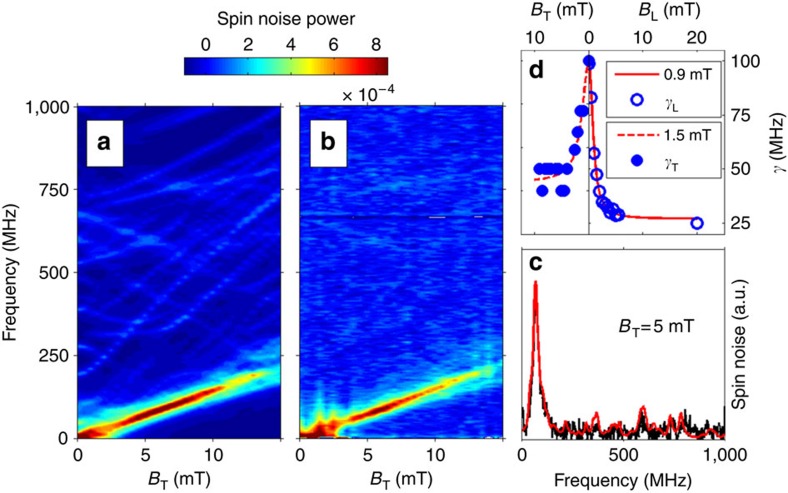
Spin noise spectra of ^55^Mn in the QW grown on CdTe versus magnetic field intensity at *θ*=30°. (**a**) Contour plot of spin noise spectra calculated for 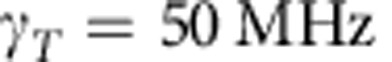, and *D*_0_=−40 neV. (**b**) Experimental contour plot (*T*=6 K). (**c**) Fitting equation (2) to the experimental spectrum determines 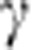. (**d**) Obtained values of 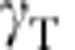 in transverse field and 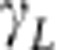 in longitudinal fields (spectra in longitudinal fields given in Supplementary Fig. 4). Red curves are fits of 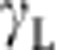 and 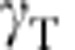 to lorentzian (HWHM=0.9 and 1.5 mT, respectively).

Including a 
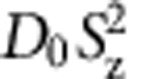
 term also in the quasi-lattice-matched QW grown on CdTe is sufficient to obtain a relatively good agreement between experiment and theory (see Fig. 3). The anisotropy coefficient *D*_0_=−40 neV is significantly larger than the calculated value *D*_0_=−5 neV. The expected fourfold cubic symmetry for magnetic fields applied in the (001) plane of the QW is observed (Fig. 4). Fig. 3a also reveals that the line centred at 75 MHz consists in many unresolved individual lines. Fig. 5 shows that this bunch of lines shifts almost linearly with the magnetic field, and can be assigned to the Zeeman transitions with *g*_*F*_=1. These lines are spread in frequency both by the crystal field, and by the quadratic Zeeman effect which arises because of the gradual decoupling of electronic and nuclear spins. The quadratic Zeeman effect becomes more visible above 10 mT, where the *g*_*F*_=1 line progressively broadens due to its splitting in many unresolved individual lines. Barely visible transitions at higher frequencies correspond to inter-hyperfine transitions Δ*F*=1. We point out that all the peaks of the unconventional spectra revealed by our low-field measurements, must evolve and contribute at high field to the well-known structure of the Mn spin resonance spectra consisting of six equally spaced lines.

### Linewidths

Our results give new insights in the Mn spin relaxation mechanisms at low magnetic field, a regime usually not accessible by conventional spin resonance techniques. In the transverse configuration, although the presence of many unresolved lines complicates the determination of the broadening parameter 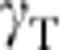, it can be estimated by fitting the experimental spectra with Equation (2) (see Fig. 5c). Fig. 5d shows that 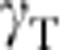 notably decreases from 100 MHz at zero field to 40 MHz above a characteristic field *B*_c_ ∼1 mT.

A potential source of broadening in this range of Mn composition *x*, is the dipolar interactions among the ensemble of electronic Mn spins^16^. Here, a Lorentzian shape is expected, with the wings of the line formed by spins with a first neighbour at short distance, and the centre due to spins with no neighbour in a volume ∼1/*xN*_0_, where *N*_0_=4/*a*^3^ is the density of cation sites in CdTe, with a cubic lattice parameter *a*=0.648 nm. Adapting the calculation of moments of ref. 17 to a Mn spin with *S*=5/2, and *g*=2, we obtain an effective field 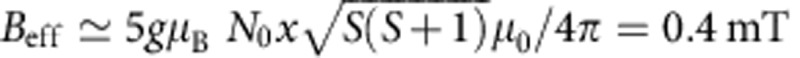. Assuming that the effect of hyperfine coupling affects the Landé factor and not *B*_eff_, the resulting linewidth with *g*=1 is 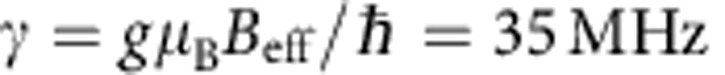, to be increased by a factor 10/3 at zero field^16^.

In a longitudinal field, 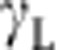 is much easier to measure, as all the intra-hyperfine lines merge at zero frequency. Spectra are given in Supplementary Fig. 3. We expect the dipolar broadening to be totally suppressed as soon as the applied field is larger than the dipolar fields. This is the case in Fig. 5d where 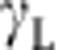 rapidly decreases; however, it saturates at a finite value, suggesting that a relaxation mechanism, with 
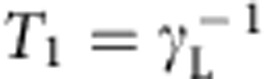
, remains to be identified.

In both cases, a simple estimation of dipolar broadening qualitatively explains the behaviour of the linewidth upon applying a magnetic field, and gives reasonable orders of magnitude. However, there is another, smaller contribution which appears to be quite independent of the applied field. Estimates of other sources of broadening are given in Supplementary Note 2. Supplementary Fig. 4 shows that the linewidth is robust to changes of temperature and excitation power.

## Discussion

As an outlook, we emphasize that the present measurements can be extended to higher magnetic fields by using large bandwidth spin noise spectroscopy techniques^18^,^19^. This will permit to study the Paschen–Back regime, when electronic and nuclear spins are completely decoupled. In this regime, because of the hyperfine interaction, the Mn electronic spin precession frequency depends on the orientation of the nuclear spin relative to the applied field^20^. The fluctuation spectra will therefore directly reveal the nuclear spin populations, an information which cannot be easily addressed by other spectroscopic methods.

The sensitivity is high enough to open the way towards the detection of a few spins in systems where the decoherence because of nuclear spins can be suppressed by isotopic enrichment^21^,^22^,^23^, and towards spin resonance microscopy with important applications in biology and materials science^24^. Additional coupling of the spins to a microcavity should enable single spin detection^10^,^25^,^26^: this will open a fascinating opportunity to explore quantum jumps of the Larmor frequency of a single Mn spin (see Supplementary Note 2), and to realize a high-fidelity readout of its nuclear spin^27^.

## Methods

### Quantum well samples

Three QW were grown by molecular beam epitaxy on (100) substrates. A 14 nm wide CdTe/Cd_0.75_Mg_0.25_Te QW was grown on CdTe as a reference: no spin noise signal could be detected for this sample without Mn. Two Cd_1-*x*_Mn_*x*_Te/Cd_0.75_Mg_0.25_Te were grown in the same conditions as the reference QW: a 14 nm wide QW grown on CdTe and a 20 nm wide QW grown on Cd_0.96_Zn_0.04_Te. Using Vegard's law and the lattice parameters of CdTe and ZnTe (ref. 28) and MnTe (ref. 29), one can expect a shear strain along the growth axis, isotropic in the growth plane, of 2 × 10^−5^, in tension, for the Cd_1-*x*_Mn_*x*_Te QW grown on CdTe and −2 × 10^−3^, in compression, for the one grown on Cd_0.96_Zn_0.04_Te. The Mn compositions, *x*=0.001, have been controlled by magneto-reflectivity. Photoluminescence revealed a trion line, likely due to residual or photogenerated holes in the QWs.

### Experimental setup

We developed a specific setup adapted to Mn spin noise spectroscopy, which requires a large bandwidth up to 1 GHz, and a high sensitivity. To that purpose we used an avalanche diode with a very low noise equivalent power (typical NEP 0.4 pW/Hz^1/2^), and a short response time of 0.5 ns. The sensitivity is then maximized by detecting the spin fluctuations in nearly crossed polarization (average power on the detector is less than ∼1 *μ*W), while keeping a relatively high probe power on the sample (typically ∼1 mW in our experiments)^30^. In these conditions, the attenuated laser is at the shot noise. Subtraction of the photon noise from the total noise is achieved by alternatively measuring the power spectra of the signal and reference beams (see Fig. 1a). The normalized spin noise power in units of the photon noise, and corrected from the apparatus response function is then given by 
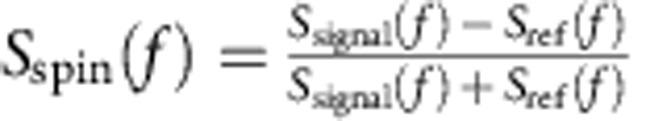
. The samples are mounted on the cold finger of a helium cryostat, and placed at the centre of two-axis Helmholtz coils. One narrowband continuous-wave diode laser (from Toptica) is tuned to the excitonic resonance of the bulk or of the QWs (see Supplementary Fig. 5). With a laser spot size ∼5 *μ*m (∼4 × 10^6^ Mn atoms lie within the spot size in the case of the QW). The spin noise signal is continuously digitized at 2 GHz and processed by a field-programmable gate array (Agilent card U1080A), to obtain the spin noise power spectrum. Typically, each spectrum requires a few minutes of signal averaging.

## Additional information

**How to cite this article:** Cronenberger, S. *et al.* Atomic-like spin noise in solid state demonstrated with manganese in cadmium telluride. *Nat. Commun.* 6:8121 doi: 10.1038/ncomms9121 (2015).

## Supplementary Material

Supplementary Information Supplementary Figures 1-5, Supplementary Note 1-2 and Supplementary References
