# Supplementary material for: Atomic-like spin noise in solid-state demonstrated with manganese in cadmium telluride
Source: Nat Commun. 2015 Sep 18;6:8121. doi: 10.1038/ncomms9121 (PMC4595592; doi:10.1038/ncomms9121)
Supplement: Supplementary Information — Supplementary Figures 1-5, Supplementary Note 1-2 and Supplementary References [file ncomms9121-s1.pdf]

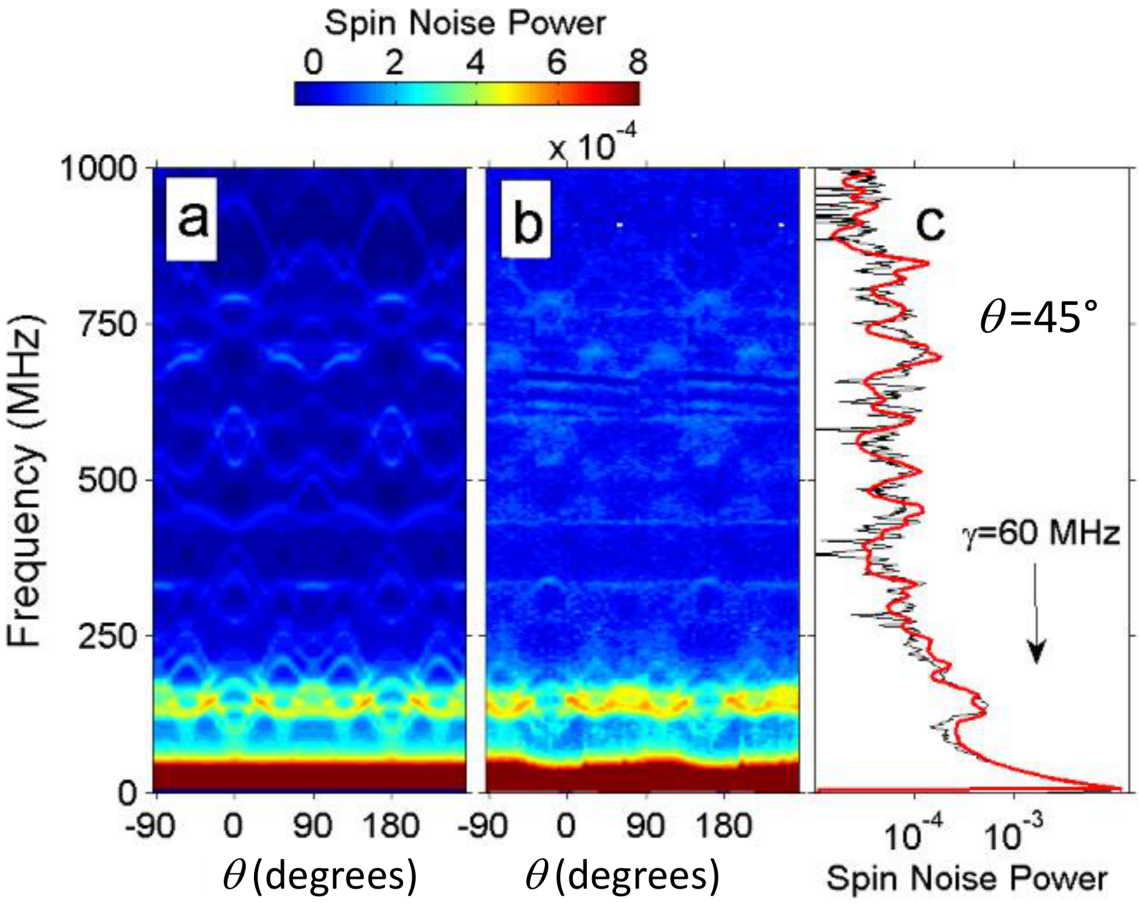

Supplementary Figure 1: **Spin noise spectra of** ^{55}**Mn in bulk sample at** B_{L}**=10.5 mT**, before subtraction of the zero-frequency line. **a**, Contour plot of the spin noise spectra calculated with Eq. (2) for $\gamma_{\mathrm{T}} = 50$ MHz, plus a Lorentzian line centered at zero-frequency with $\gamma = 60$ MHz. θ is the angle between magnetic field and the [001] direction in the (110) plane **b**, Contour plot of the experimental spectra measured at T=4.8 K and laser power P=1.2 mW. **c**, Experimental (black curve) and theoretical spectra (red curve) at $\theta = 45°$. The origin of the zero-frequency line is not understood, but it was not observed in a CdTe QW without Mn and its broadening is very close to that of the other Mn lines. This suggests that it is also related to fluctuations of some Mn spins which do not precess, as it would be the case for strongly anisotropic Mn spins locked onto some crystallographic direction, with a point symmetry lower than the cubic symmetry of the bulk. As our Kerr rotation experiment is mainly sensitive to Mn atoms close to the surface, one possibility would be that one oxygen substitutes a tellurium in one of the Mn-Te bonding, due to surface oxidation. To confirm this hypothesis, further investigations at higher fields are necessary, particularly to determine the critical field at which the spins unlock.

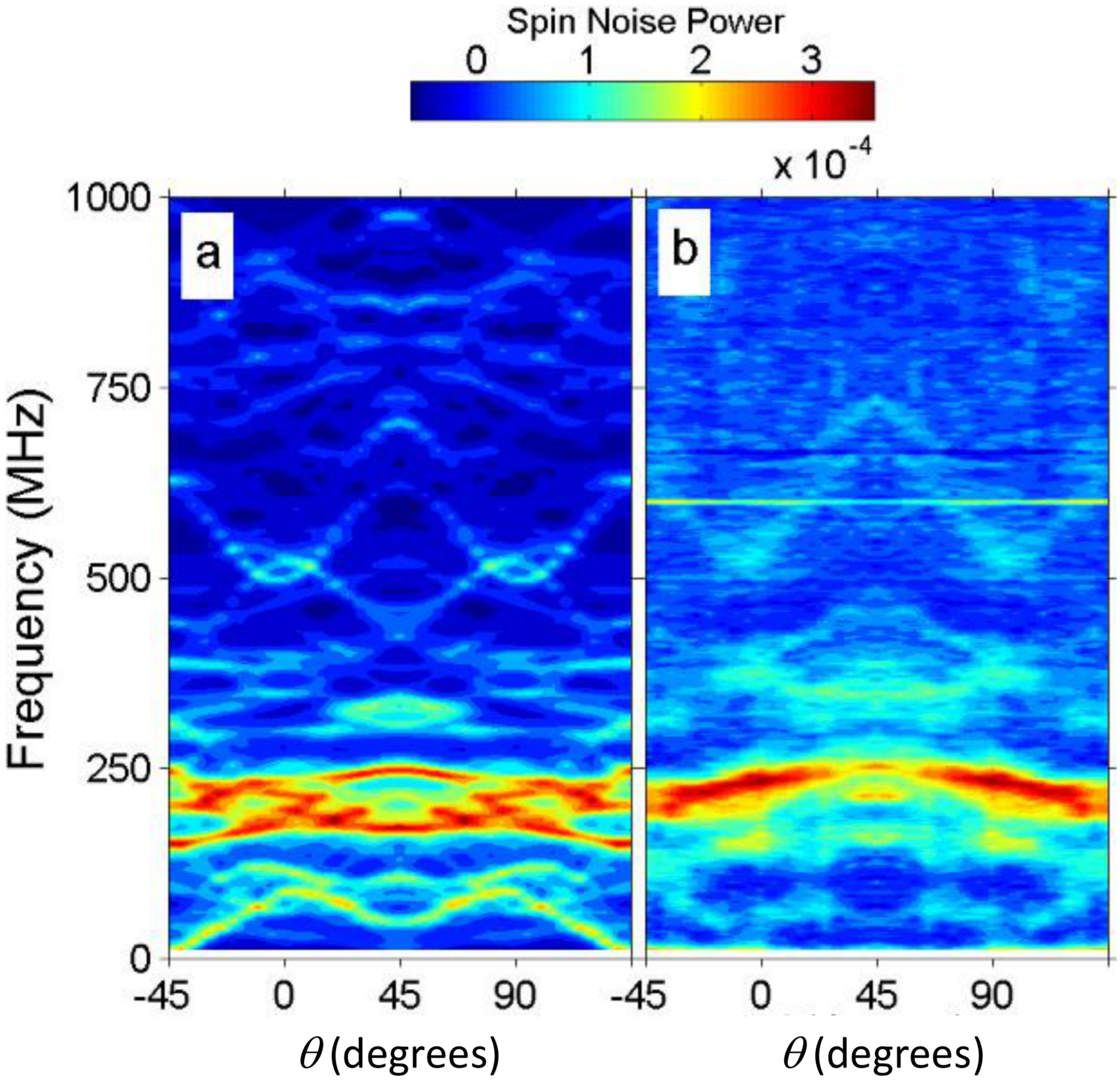

Supplementary Figure 2: **Angular resolved spin noise spectra at B_{T}=12 mT in the QW grown on CdZnTe substrate.** **a**, Contour plot of spin noise spectra calculated for anisotropy parameters $D_0 = 473$ neV and $E_0 = -30$ neV, and $\gamma_{\mathrm{T}} = 50$ MHz. θ is the angle between the magnetic field and the [100] axis in the (001) plane. **b**, The experimental contour plot (at T=5 K and P=1.2 mW) clearly reveals that the [110] and [1-10] axes are not equivalent. Due to the biaxial strain induced by the lattice mismatch, a spin anisotropy with $D_0 \simeq 0.47\mu$eV is expected. However the breakdown of the fourfold symmetry reveals the existence of at least an uniaxial strain in the QW plane parallel to a [110] direction, described by a new term $E_0(S^2_{[110]} - S^2_{[1\text{-}10]})$ in the spin hamiltonian. Some agreement with experiment was obtained for $E_0 = -30$ neV; nevertheless the spin hamiltonian with biaxial and uniaxial strains fails to reproduce correctly the spin noise spectra, especially below 250 MHz where most of the spin noise power is concentrated. Solving this puzzle will require more systematic studies on strained samples.

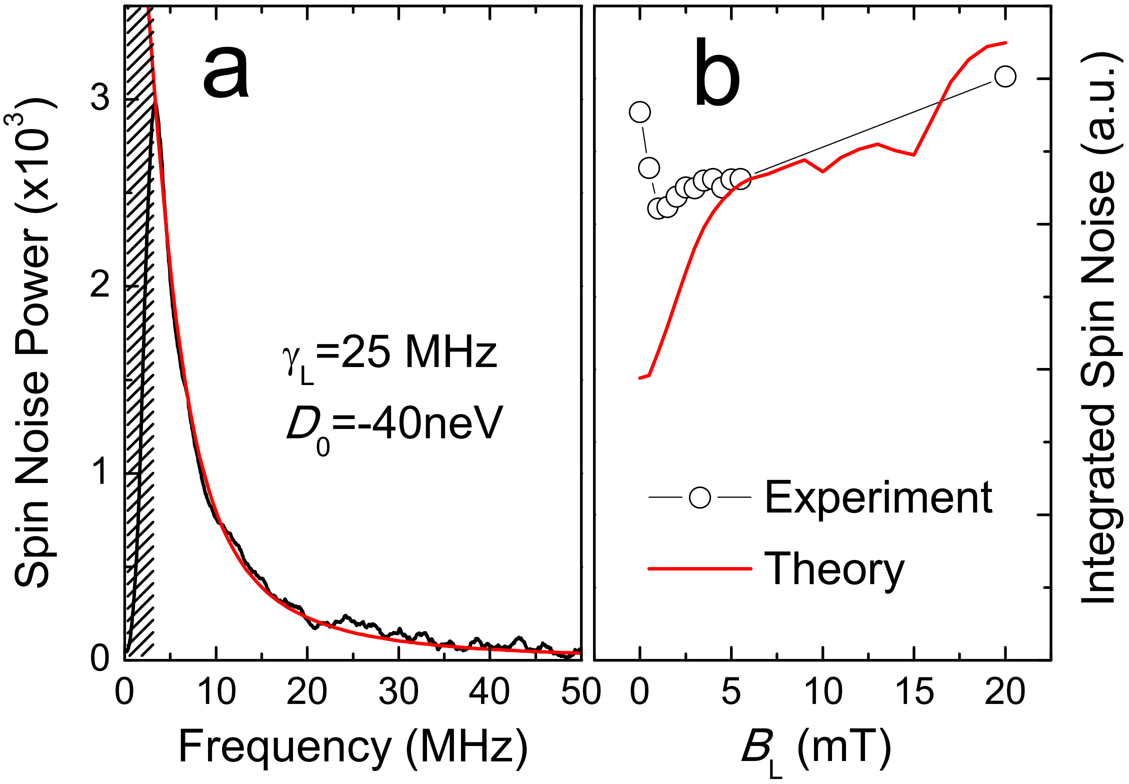

Supplementary Figure 3: Low-frequency spin noise of ^{55}Mn in longitudinal field, spectra at T=3.7 K from a QW grown on CdTe substrate with the field applied along the normal to the sample and the direction of light propagation. **a**, Low-frequency spin noise spectrum at B=5 mT. The theoretical spin noise spectrum (red) is a fit to the data, which determines γ_L = 25 MHz. The cutoff in the experimental spectrum (black) below 3 MHz (hatched region) is due to the detector response. **b**, Integrated spin noise power of the zero-frequency line from 0 to 50 MHz. A twofold increase of the integrated noise power with magnetic field is predicted by the theory (red solid line). Although γ_L is found to vary from 100 MHz to 25 MHz (see Fig. (5d) of the paper) for simplicity it is fixed at 50 MHz in the present calculation, but this does not change significantly the integrated spin noise (the HWHM is given by γ_L/π and does not exceed 33 MHz). As the field increases the spin projection along the field becomes a good quantum number. Therefore all inter-hyperfine matrix elements progressively vanish and the noise power concentrates in the zero-frequency line: this is confirmed by the experimental data (symbols) above 5 mT. At lower fields, the noise power first decreases contrary to theoretical expectations.

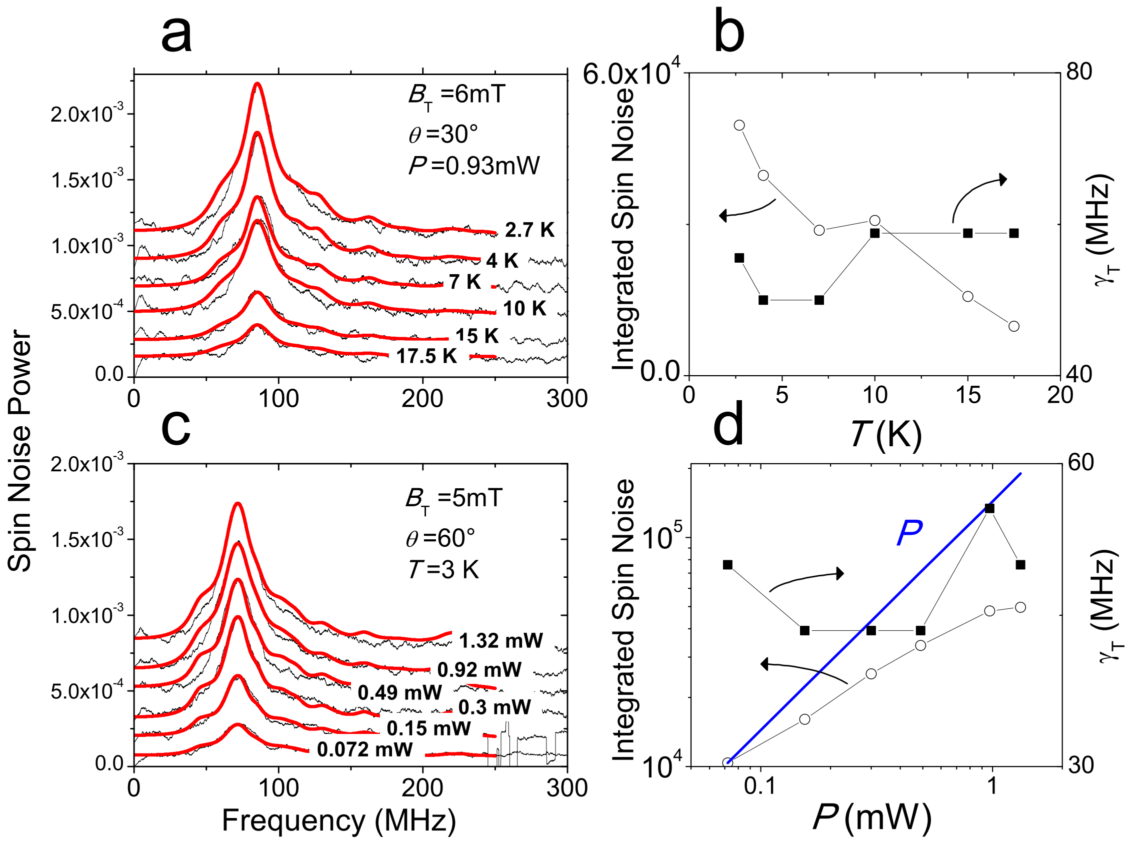

Supplementary Figure 4: Spin noise versus temperature and laser power - QW on CdTe substrate a, black solid lines are spin noise spectra measured at different temperatures. Eq. (2) of the article is fit to the $g = 1$ line with γ_T being the only fitting parameter. Other lines are too weak to reliably determine their linewidth. At each temperature the probe wavelength was tuned to follow the excitonic resonance: one observes a rapid decrease of the integrated spin noise with temperature. As the Mn spins are paramagnetic and weakly interact with each other, their susceptibility scales inversely with temperature. From the fluctuation-dissipation theorem the integrated spin noise should be proportional to the susceptibility times the temperature, and thus constant. Therefore, the strong decrease of the Kerr rotation noise power is not due to a decrease of the spin noise power, but rather to a decrease of the magneto-optical response of the QW.**b**, Integrated spin noise power, and broadening γ_T of the $g = 1$ line, versus temperature. γ does not vary significantly up to 17.5 K, and corresponds to a Mn spin relaxation time in the $17 - 20$ ns range. **c, d**, Same as **a, b** but for power series. No significant change of γ is found when the probe power increases: this shows that the Mn spin coherence is robust against the injection of photocarriers in the QW. The sublinear increase of the integrated spin noise power is probably a consequence of heating, which tends to decrease the spin noise as described above.

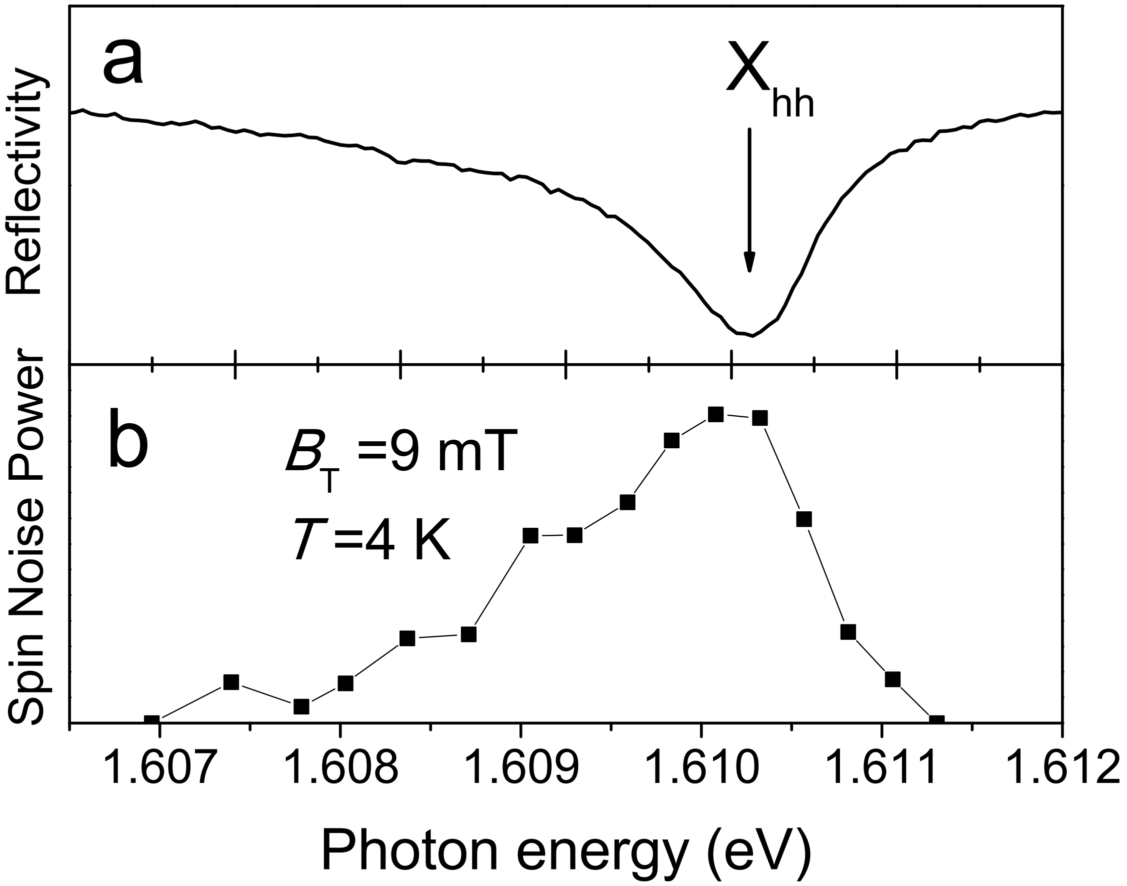

Supplementary Figure 5: Reflectivity and integrated spin noise power versus photon energy - QW on CdTe substrate a, Reflectivity spectrum. The dip is assigned to the heavy-hole exciton (X_{hh}) resonance of the QW **b**, Integrated spin noise power. The spin noise power is maximum for the photon energy resonant with the excitonic transition of the QW. This confirms that the Mn spin fluctuations are indirectly detected via the induced excitonic spin splitting.

Supplementary Note 1. Sum rules for the spin noise power.

We show here that at high temperatures, the integrated spin noise is a constant, and that, in the case $S = I$, half of the spin noise power is concentrated in the $g = 1$ line. Introducing the high temperature limit into Eq. (2) of the paper one can write $\rho_n = \rho_m = [(2S+1)(2I+1)]^{-1}$, and the integrated spin noise as

$$\int_{-\infty}^{+\infty} (\mathbf{S}\cdot\hat{\alpha})^2_\omega d\omega = \frac{2\pi}{(2S+1)(2I+1)} \sum_{n,m} \langle n|\mathbf{S}\cdot\hat{\alpha}|m\rangle\langle m|\mathbf{S}\cdot\hat{\alpha}|n\rangle \tag{1}$$

Using the closure relation $\sum_m |m\rangle\langle m| = 1$ and the invariance of the trace one obtains the sum rule

$$\int_{-\infty}^{+\infty} (\mathbf{S}\cdot\hat{\alpha})^2_\omega d\omega = \frac{2\pi}{3} S(S+1) \tag{2}$$

which is isotropic. Then we calculate the integrated spin noise, restricted to the $g = 1$ line. This line corresponds to the slow precession of the total angular momentum $\mathbf{F} = \mathbf{I} + \mathbf{S}$ in the external field, while $\mathbf{I}$ and $\mathbf{S}$ rapidly precess around each other. This means that S is aligned along F, and one can write $\mathbf{S} = C_F\mathbf{F}$, C_F being a constant (more rigorously, this is a consequence of the Wigner-Eckart theorem). Squaring both side of $\mathbf{I} = \mathbf{F} - \mathbf{S}$ gives $I(I+1) = F(F+1) + S(S+1) - 2\mathbf{S}\cdot\mathbf{F}$, which in the case $S = I$ gives $\mathbf{S}\cdot\mathbf{F} = F(F+1)/2$, hence $C_F = 1/2$. The spin noise power integrated over the $g = 1$ line becomes

$$\int_{-\infty}^{+\infty} (\mathbf{S}\cdot\hat{\alpha}|_{g=1})^2_\omega d\omega = \frac{\pi}{2(2S+1)^2} \sum_{F=0}^{2S} \sum_{M=-F}^{+F} \langle F,M|(\mathbf{F}\cdot\hat{\alpha})^2|F,M\rangle \tag{3}$$

A straightforward calculation finally gives

$$\int_{-\infty}^{+\infty} (\mathbf{S}\cdot\hat{\alpha}|_{g=1})^2_\omega d\omega = \frac{\pi}{3} S(S+1) \tag{4}$$

which is also isotropic, and shows that half of the spin noise power is concentrated in the $g = 1$ line.

Supplementary Note 2: Broadening mechanisms and prospect on the nuclear spin relaxation and the observation of quantum jumps.

Several possible mechanisms of inhomogeneous and homogeneous broadening can be discarded.

One possible source of inhomogeneous broadening is the eventual existence of a strain distribution. This is however very unlikely in the bulk and in the QW grown on CdTe, because the average strain is quite small. The exchange field created by the polarized resident carriers in the QW may slightly shift the Mn precession frequency depending on their position inside the QW, an effect similar to the Knight shift for the nuclei [1]. In this case the broadening should increase with the magnetic field, in contradiction with our results. Finally, each Mn atom feels a different quasi-static nuclear (superhyperfine) field created by its Cd neighbors. This field, of the order of few Gauss [2], randomizes the Mn spin orientation on a time scale ~ 100 ns.

Next, we consider homogeneous broadening mechanisms. Because of the dipole-dipole interactions between the fraction of nuclei which have a nuclear spin, the nuclear field fluctuates on a time scale ~ 10 ms [3]. Therefore the superhyperfine field acting on the Mn electronic spin also fluctuates on this time scale, which is much larger than the Larmor period of the Mn spins in the nuclear field. In this regime the electronic spins follow adiabatically the nuclear field, so that their relaxation time ~ 10 ms. Spin-lattice relaxation at low temperature and concentration is of the order of tens of milliseconds [4]. Relaxation by free electrons is in the microsecond range for our estimated carrier concentration $n = 10^{10}$ cm^{-2} [5].

As none of these mechanisms can account for the observed linewidth of our spin noise spectra, we are left with the electronic dipole-dipole interaction discussed in the paper.

The above discussion on the identified Mn spin relaxation mechanisms may serve as a basis to predict the ultimate limit for the nuclear spin relaxation times. Most of these mechanisms can be suppressed by isotopic purification, control of the residual concentration of free carriers, and increased dilution of magnetic atoms. We will be left with the spin-lattice relaxation of isolated Mn in the host lattice. At low temperature the electronic spin relaxation is due to one-phonon processes, and was calculated by Blume and Orbach [6]. In a magnetic field $B = 0.3$ T, high enough to decouple electron and nuclear spins (Paschen-Back regime), and a temperature $T = 4$ K, we get $T_{1e} \sim 0.2$ s. In an isotopically purified host, and at very low Mn concentration, the nuclear spin relaxation will be dominated by the fluctuating hyperfine field created by the $3d$ electronic shell, with a correlation time $\tau_c = T_{1e}$. The nuclear Larmor frequency ω times τ_c largely exceeds unity, so that in this limit the nuclear coherence time $T_{2n} \simeq \tau_c \sim 0.2$ s. More precisely it can be shown that $T_{2\mathrm{n}} = 2T_{1\mathrm{e}}$ [7]. The longitudinal nuclear spin relaxation varies like $T_{1\mathrm{n}} = T_{2\mathrm{n}}(1+(\omega\tau_c)^2)$. As $\omega\tau_c \sim 10^6$, the relaxation by the hyperfine interaction is nearly completely suppressed, because the energy is not conserved during a mutual electron-nucleus spin-flip. Under these conditions the Mn nuclear relaxation is rather limited by the direct nuclear spin-lattice interaction even if it is a very inefficient process: as an example nuclear spin-lattice relaxation takes 200 hours at 77 K in silicon [8]. Observation of quantum jumps of the electronic Larmor frequency would give a direct determination of the Mn nuclear spin-lattice relaxation time.

Supplementary References.

[1] Vladimirova, M. *et al.* Dynamics of the localized spins interacting with two-dimensional electron gas: coexistence of mixed and pure modes. *Phys. Rev. B* **78,** 081305 (2008).
[2] Lambe, J. & Kikuchi, C. Paramagnetic resonance of CdTe : Mn and CdS : Mn. *Phys. Rev.* **119,** 1256 (1960).
[3] Meier, F. and Zakharchenya, B. , eds., *Optical Orientation*, Modern Problems in Condensed Matter Science Series **8,** North-Holland, Amsterdam (1984).
[4] Scalbert, D., Cernogora, J. & Benoit a La Guillaume, C. Spin-lattice relaxation in paramagnetic CdMnTe. *Solid State Commun.* **66,** 571 (1988).
[5] Dyakonov, M., ed. *Spin Physics in Semiconductors*, Springer, Berlin (2008)
[6] Blume, M. & Orbach, R. Spin-lattice relaxation of S-state ions: Mn^{2+} in a cubic environment. *Phys. Rev.* **127,** 1587 (1962).
[7] Morton, J. J. L. *et al.* Solid-state quantum memory using the 31P nuclear spin. *Nature* **455,** 1085-1088 (2008)
[8] Lampel, G. Nuclear Dynamic Polarization by Optical Electronic Saturation and Optical Pumping in Semiconductors. *Phys. Rev. Lett.* 20, 491 (1968).
